# The Seroprevalence of Human Cystic Echinococcosis in Iran: A Systematic Review and Meta-Analysis Study

**DOI:** 10.1155/2016/1425147

**Published:** 2016-10-17

**Authors:** Reza Shafiei, Saeed Hosseini Teshnizi, Kurosh Kalantar, Maryam Gholami, Golnush Mirzaee, Fatemeh Mirzaee

**Affiliations:** ^1^Vector-Borne Diseases Research Center, North Khorasan University of Medical Sciences, Bojnurd, Iran; ^2^Clinical Research Development Center of Children Hospital, Hormozgan University of Medical Sciences, Bandar Abbas, Iran; ^3^Department of Immunology, School of Medicine, Shiraz University of Medical Sciences, Shiraz, Iran; ^4^Rehabilitation Management, University of Social Welfare and Rehabilitation Sciences, Tehran, Iran; ^5^School of Nursing and Midwifery, Shahrekord University of Medical Sciences, Shahrekord, Iran

## Abstract

Human cystic echinococcosis (HCE), a zoonotic infection of the larval stage of* Echinococcus granulosus*, has high effect on public health in human population all around the world. Iran is one of the most important endemic areas in the Middle East. This systematic review and meta-analysis was performed to evaluate the seroprevalence of HCE in Iranian population. An electronic search for articles from 1985 until April 2015 was performed using data bases PubMed, Scopus, Google Scholar, Magiran, IranMedex, Iran Doc, and Scientific Information Database (SID) both in English and in Persian. A random-effects meta-analysis was used to combine results from individual studies. The information was analyzed by STATA version 11.1. A total of 33 articles met our eligibility criteria and were included in a meta-analysis. The pooled estimate of the prevalence of HCE based on random-effects model was estimated 6.0% (95% CI: 4.0%, 7.0%). The prevalence of the disease significantly increased with age and prevalence rate in males was significantly lower than females (*p* < 0.001). The using of CIE or CCIEP method was also significantly greater than the other methods (*p* < 0.001). There was a publication bias in prevalence of studies. HCE is highly prevalent in Iran. Public education for preventive strategies and finally reducing transmission of the parasite and infection in population is needed.

## 1. Introduction

Human hydatidosis or human cystic echinococcosis (HCE) is a chronic parasitic infection disease caused by the larval stage of* Echinococcus granulosus* which has an important effect on public health in human populations [1, 2]. This zoonotic disease is initiated by accidental ingestion of the parasite's egg. It can be transmitted by infected feces of dogs via soil, vegetables, contact with dog, water, food, and so forth [3, 4].

The disease was widely distributed mostly in regions where sheep-rearing is a major industry [5, 6]. This multihost disease is one of the most important public health infection diseases in Iran [6, 7]. Echinococcosis is one problem not only in humans but also in traps. It causes a huge economic burden for governments. That is why Ministry of Health and veterinary organizations pay more attention to it every year and have regular instruction programs for prevention of this infection. For example, they kill wild dogs; they also prescribe antihelminthes drugs in pet dogs and train the people to wash vegetables. The prevalence of hydatidosis in dogs has been recorded to be 5–45% in Iran [8]. The infection rate with different strains of* E. granulosus *sensu lato in various domestic livestock has been reported to be 24.41%, 8.51%, 18.89%, 35.76%, and 35.21% in sheep, goat, cattle, buffalo, and camels, respectively [8–10]. The incidence of surgical cases of HCE is estimated to be 1.18–3 per 100,000 in different medical centers of Iran provinces and territories [3].

Early diagnosis of HCE is difficult due to being asymptomatic in early stages while using physical imaging; particularly ultrasound (US) examination is helpful not only at late stages but also for early diagnosis (cysts under 1 cm in diameter) [2, 11]. The early diagnosis of HCE is based on available immunodiagnostic techniques with specific immunodominant antigens such as Ag B and US imaging. Methods for detecting specific antibodies can provide opportunities for early treatment of the disease [11–13]. Immunodiagnostic techniques have been used for total screening of population in endemic regions but the sensitivity and specificity of the diagnostic antigen are important [11, 12]. Several immunochemical tests such as ELISA and IFA are developed for determining anti-echinococcus IgG in serum for the diagnosis of HCE in Iran.

Given the high prevalence and the greater importance of this parasite, as well as the economic losses and the significant mortality and morbidity that HCE has in Iran, and due to the fact that the prevalence of this disease has not been identified in Iran yet, the need for a comprehensive study with the aim of serological monitoring of this disease in Iran seemed necessary. In this systematic review and meta-analysis study, we provide some insights into seroprevalence of HCE in different provinces of Iran from 1985 to 2015 where noncoordinated mass screenings have been performed in the past.

## 2. Methods

### 2.1. Search Method

The medical publications in English and Persian electronic databases were searched including PubMed Medical Subject Headings (MeSH/mh), Google Scholar, Magiran, Iran Medex, Iran Doc, and Scientific Information Database (SID) both English and Persian databases from 1985 to April 2015. Publication searches were applied using the keywords: “Seroprevalence”, “Serological prevalence”, “Cystic Echinococcosis”, “Hydatid cyst”, “Hydatidosis”, “Human”, “IgG antibody”, and “Iran” in combination or alone. To reduce the possibility of selection bias in this study, criteria were clearly defined and studied.

### 2.2. Data Extraction

The information was extracted from the included studies using a standard form by the two independently reviewers (RSH, STH). Any disagreement was resolved by discussion between the two reviewers. If consensus could not be reached, a third reviewer was consulted (MGH). Kappa index showed an agreement of 89% between the two reviewers.

The standard form consisted of the following variables: first author; year of publication; location, sample size, positive subjects, age and sex of participants, and lab methods. The outcome was the prevalence of seroprevalence and this was obtained for each study by dividing the number of positive cases to the total sample size.

### 2.3. Statistical Analysis

In this meta-analysis study outcomes were the prevalence of the seroprevalence of HCE. Forest plot was used to visualize the heterogeneity among studies. The results for each study and pooled outcome were revealed as a forest plot [reported as effect size (ES) with a 95% confidence interval (CI)]. Cochran's heterogeneity statistic or *Q*-test (*p* < 0.1 indicated heterogeneity) and *I* ^2^ statistic were used to examine the difference in study variability due to heterogeneity rather than chance, with a range from 0 to 100 percent (values of 25%, 50%, and 75% are considered to represent low, medium, and high heterogeneity, resp.). Subgroup meta-analysis analysis was used to compare the prevalence of hydatidosis among age, sex, and lab methods groups. Egger's test was used to evaluate the publication bias [14, 15]. Statistical analyses were conducted using the Statistical Software Package (STATA) version 11.1.

## 3. Results

**Figure 1:**
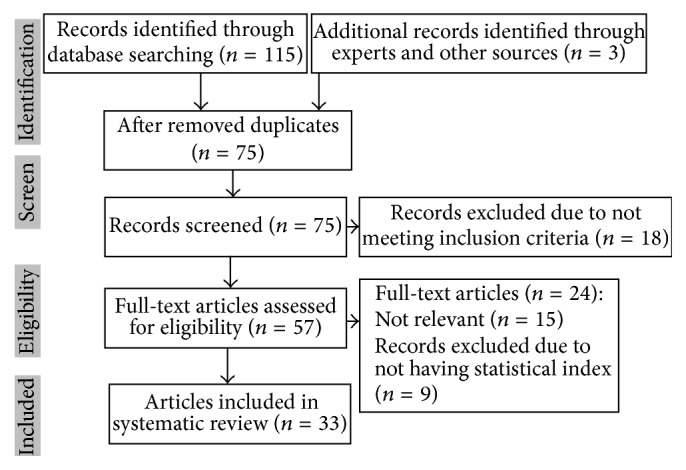
PRISMA flowchart describing the study design process.

Our initial database searches identified 115 articles and an additional 3 studies through hand searches and expert suggestions, giving a total of 118 articles that were screened. Out of these, 57 were chosen for reading of full text and 33 were included in this meta-analysis.  Figure 1 shows the diagram of article selection according to the PRISMA statement.

**Figure 2:**
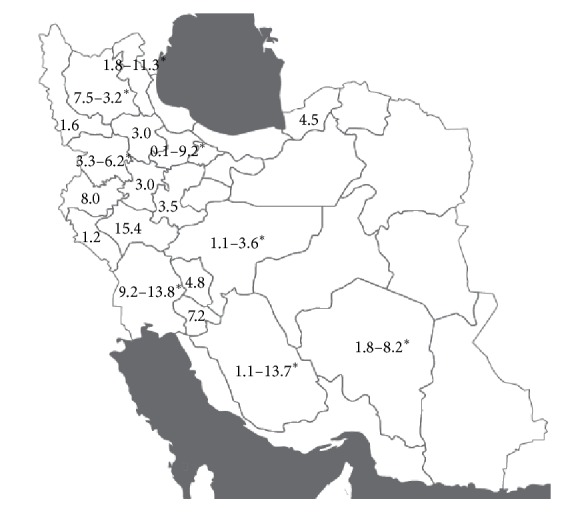
Graphic representation of prevalence (%) of HCE in provinces in Iran. The asterisk (*∗*) means the minimum and maximum of prevalence in province.

Generally, the most prevalence of this disease took place in the western and southwestern areas of Iran and the highest prevalence was related to Lorestan, Fars, and Khuzestan provinces and the lowest rate was related to Tehran (Figure 2).

From 42706 people 2551 were positive for anti-echinococcosis. Indirect fluorescent antibody (IFA), enzyme-linked immunosorbent assay (ELISA), counterimmunoelectrophoresis (CIE) or counter-current immunoelectrophoresis (CCIEP) were used in all of the studies. ELISA was the mostly used test in this study, 17 (51.52%). Target group of most of studies was healthy volunteers 24 (72.72%) (Table 1).

**Figure 3:**
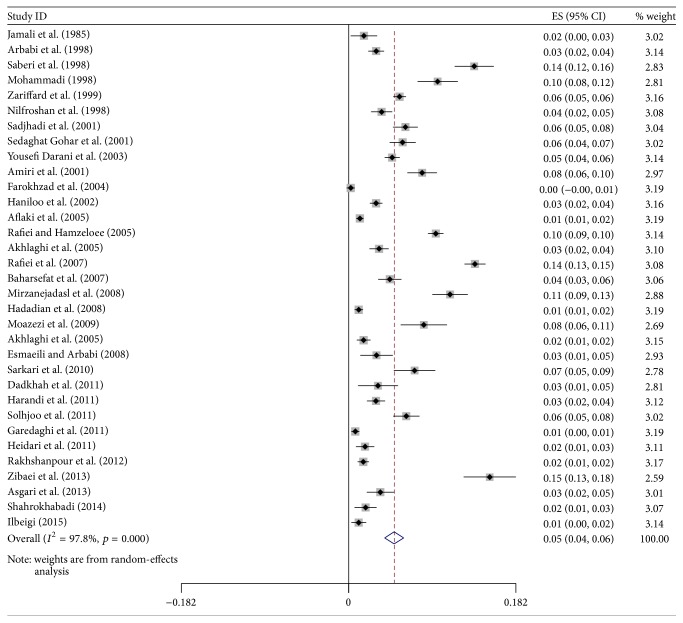
Forest plot of meta-analysis results for prevalence of CE. The middle point of each line indicates the prevalence rate and the length of line indicates 95% confidence interval of each study. Rhombus shape indicates 95% confidence interval for all studies.

There was a strong heterogeneity in the prevalence of the studies (*I* ^2^ = 98%, *p* < 0.0001). The pooled estimate of the prevalence of HCE based on random-effects meta-analysis was obtained 5.0% (95% CI: 4.0%, 6.0%). The highest prevalence was related to study of Zibaei et al. (2013) with prevalence 15.4% in Khorramabad province [16] study carried out by ELISA and the lowest prevalence was 0.2% in Farrokhzad et al. (2004) in Tehran study carried out by IFA [17]. Also the pooled prevalence significantly was higher than zero line (ES = 0: *z* = 10.03, *p* < 0.001) (Figure 3).

The subgroup analysis indicated that the prevalence of HCE was significantly increased with the increase of age (*p* < 0.001). Also prevalence of CE among males, 2.1 (95% CI: 1.8%, 2.4%), was significantly lower than females, 3.6% (95% CI: 3.2%, 3.9%) (*p* < 0.001). Also the prevalence for CIE method was significantly greater than other methods (*p* < 0.001) (Table 2). The results of Egger's test showed there was not a publication bias among studies (coef. = −0.011, SE = 0.008, *p* = 0.326). Therefore, this meta-analysis included two types of studies, high and low prevalence of HCE.

## 4. Discussion

HCE as an emerging neglected disease is a major public health problem in many countries which results in substantial economic resource loss [5]. The mentioned disease has a global distribution with an annual occurrence ranging from 1 to 200 per 100,000 individuals [1]. The prevalence rate of HCE based on hospital cases is different in Iran with rate of >1% of total population [7, 18] and this is the most commonly used index of HCE [16]. Most of the infected people with the larva of* E. granulosus* have a delay in showing related symptoms such as cyst-like mass which grows gradually among various groups [5]. So serological examination alone is useful for giving an approximate evaluation of the infection pressure and might be useful (used on already collected serum samples, such as those found in blood banks) to provide data on the level of presence of* E. granulosus* in a given area to identify asymptomatic cyst carriers generally. We must also have plans to control this disease. To achieve a more accurate diagnosis, mass screening should include both ultrasound examination and serology [19].

The result of our investigation showed that the range of HCE is 0.2% in Tehran with IFA by Farrokhzad et al. (2004) [17] while Zibaei et al. recorded a higher seroprevalence (15.4%) in Khorramabad in the southwest of Iran with ELISA [16]. The prevalence of HCE infection in this survey was higher in southwest and south of Iran with 13.7% by Rafiei et al. (2007) in Khouzestan [20] and Saberi-Firouzi et al. (1998) in Fars [21]. The higher prevalence found by the studies which used counterimmunoelectrophoresis (CIE) or counter-current immunoelectrophoresis (CCIEP) may seem paradoxical since this test is usually considered to be more specific than sensitive (it is used as a confirmation test in some countries; it is time-consuming and is rarely used as a screening test) [18]. Besides this test, ELISA with B antigen was used in most of the studies (20 studies) because this method is acceptable, easy, efficient, and affordable and has a high level of sensitivity and specificity. In addition, since preparing the B antigen is easy, using local area antigen shows highly accurate test results. Applying of different kinds of serological methods could be the reason for controversy of obtained results. In addition, it seems that using the serological methods with high sensitivity and specificity would be helpful and something must be noticed that for each experiment in each part of world it is highly recommended to use the antigen which is prepared from that area. Thus, using the antigen which is from a specific district should be used in ELISA test. This strategy could have made the ELISA test more reliable in comparison to those tests which are using the universal antigen. So, we recommend that ELISA test by means of local antigens could help us to get rid of the controversy results obtained from different groups [9].

Table 1 and Figure 2 show the model studies in the country and the seropositivity rate in patients. As it can be seen, no studies have been performed in the eastern and central parts of Iran and the reason is the low prevalence of the disease especially in southeastern areas of the country. The eastern and central parts of Iran do not have enough pasture. That is why it is not a good place for animal husbandry, and consequently there are not enough intermediate hosts for this kind of helminthes. Furthermore, the parasite egg is sensitive to high temperature and low humidity. So, on one hand lack of intermediate host for this worm and on the other hand existing industrial abattoir make the life cycle of such helminthes unfinished because the infected meat of intermediate hosts is not available for the final host. Based on the mentioned reasons we have less infected people with HCE. That is why we have a small number of infected people who do surgery to remove cysts. This issue makes the seroepidemiological study difficult because of the lack of samples [21]. On the other hand, the highest prevalence rate of this parasite belongs to the western parts of the country especially in the provinces of Lorestan, Kohgiluyeh and Boyer-Ahmad, Khuzestan, Fars, and Ardabil which have a mild climate and more rainfall and humidity. This fact consequently increases the survival rate of the eggs and the transmission cycle of the parasite. Therefore, the different climate conditions of the country have an effective role in the infection prevalence rate. On the other hand, due to the good weather conditions, the rate of livestock raising and grazing is very high in these areas which can cause parasite infection in the intermediate hosts and eventually in the final hosts. It must be noted that the stray dogs which are infected with the parasite play a very big role in spreading the disease and increasing the prevalence rate in these areas [11].

On average, the hydatidosis rate in stray dogs is 5–94% in different regions of Iran [8] and the median rate of infection in the stray dogs is 20% in the western areas [9] which shows a high rate of infection in the final hosts. One more thing to be kept in our mind is about cultural and religious issues existing in Iran. Here in Iran in the rural parts, most of the people have dogs and these dogs are vaccinated and cannot consider them as a source of infections [3]. These infected stray dogs can easily spread large numbers of parasite eggs on the agricultural fields where vegetables grow. This is considered as one of the main factors in increasing the prevalence of the disease in intermediate hosts including humans in these regions. Also the human behavior in contact with dogs plays an important role in the transmission of infection in humans. This behavior is closely related to peoples' cultural and economic conditions. Because people who keep dogs in these areas are more sensitive to the dogs' infections, these people are also inferior to other people in terms of educational and economic conditions [8].

This study identified that the prevalence of HCE infection was usually higher in rural inhabitants rather than in urban ones [16, 19, 22–26]; also most of the studies showed that females were the main subject of HCE [9, 20, 22, 25, 27–31]. One evidence of this result is that most farmers and housewives that come in contact with infection source are females that live in rural areas [8]. In addition, the presence of an unlimited dog population in rural communities contributes to the exposure to* E. granulosus* and high seroprevalence rate. Slaughtering practices (noncontrolled family slaughtering in rural areas versus more publicly or privately organized and controlled slaughtering in cities, especially Tehran) and permanent presence of intermediate hosts (sheep) to maintain the cycle are important too [5].

The results of our investigation showed that prevalence of HCE is high among subjects in the middle-aged people in most of the studies. They reported the age range of 10–19 as the highest infected age group in Zanjan [29], 20–40 age range in Kurdistan [32], 60–69, 60–80, and 60–90 age range in Isfahan, Hamadan, and Ardabil [18, 26, 33], 20–29 years old in Khorramabad [16], 30–39 years old in Yasuj [24], and 29–39, 40–49, and 30–60 years old in Kerman, Arak, Golestan, and Qom, respectively [19, 34–36].

These heterogeneity differences seen in the studies performed in different regions of the country are due to the classification of different age groups, the difference in the type of the studied people, and the geographical location of each study. Also long patent period of this disease is one of the problems [18]. The issue of age groups also includes the population's access to care: if diagnosis is made rather early because of easy availability of US examination and if young people are operated on because of the proximity of well-equipped hospitals and of surgeons ready to work (and earn their living!), there will be higher prevalence in older age groups. As different studies show different results and with most cases being diagnosed many years after their infection, the detection of true age group in the context of infection with HCE is more difficult. Mass screening has well shown that the majority of cysts will remain asymptomatic and even spontaneously degenerate in the majority of subjects; serology does not distinguish between rapidly progressing cases and stable or even aborted cases. Several publications support this [11].

### 4.1. Strengths and Weaknesses of Study Designs

These studies demonstrate the importance of serologic HCE in various groups of people in Iran. The control of the disease in Iran where most dogs are stray and are infected with adult worms all around the cities is difficult. Therefore, public education that highlights the importance of washed vegetables and inhabitation of exposure to the source of infection could reduce the transmission of the parasite and the consequences of infection in humans [6, 13].

In general, performing such systematic studies on the rate of the human hydatid cysts seroepidemiology in different parts of the world especially in parasite endemic countries like Iran which is located in the Middle East can show the general trend of the disease in its different parts. Studying the general pattern of the disease in different regions can help a lot in planning health and disease prevention programs. Considering the very high economic losses in terms of mutilating the infected organs of the domestic animals as well as the various surgeries performed on the infected humans existing in the country, these control programs can decrease the general trend of the disease.

## 5. Conclusion

HCE is highly prevalent in Iran and could be a cause of considerable health problems in the country. Educational programs, serological screening, and the continuation of the treatment of the patients when possible could help reduce the national impacts of the disease. Further studies are needed to describe the exact epidemiology of the disease at a national level in other parts of Iran.

## Figures and Tables

**Table 1:** General characteristics of studies included in the present systematic review and meta-analysis.

Author [reference]	Year	Province	sample	Positive (*n*)	Positive % (95% CI)	Lab method (Ag)	Target group
Jamali [37]	1995	Uromieh	300	5	1.67 (0.22, 3.12)	IFA	Villagers
Arbabi [18]	1998	Hamedan	1530	46	3.01 (2.15, 3.86)	IFA	Healthy volunteers
Saberi-Firouzi [21]	1998	Fars	1000	137	13.70 (11.57, 15.83)	CIE	Healthy volunteers
Mohamadi [38]	1998	Tehran	700	68	9.71 (7.52, 11.91)	IFA	Healthy volunteers
Zariffard [39]	1999	Western Iran	4138	230	5.56 (4.86, 6.26)	ELISA	Healthy volunteers
Nilfroshan [40]	1998	Esfehan	1000	36	3.60 (2.45, 4.75)	IFA	Healthy volunteers
Sadjjadi [13]	2001	Shiraz	1227	76	6.19 (4.85, 7.54)	CCIEP	Healthy volunteers
Sedaghat Gohar [27]	2001	Tehran	1052	62	5.89 (4.47, 7.32)	IFA	Healthy volunteers
Yousefi Darani [28]	2003	Chaharmahal	2524	120	4.75 (3.92, 5.58)	CIE	Surgical patients
Amiri [41]	2001	Kermanshah	1072	86	8.02 (6.4, 9.65)	IFA	Patients and blood donors
Farrokhzad [17]	2004	Tehran	437	1	0.23 (−0.22, 0.68)	IFA	Healthy volunteers
Haniloo [29]	2002	Zanjan	2367	71	3.00 (2.31, 3.69)	ELISA	Healthy volunteers
Aflaki [22]	2005	Eilam	3000	37	1.23 (0.84, 1.63)	Dot-ELISA	Healthy volunteers
Rafiei [42]	2005	Khuzestan	4596	437	9.51 (8.66, 10.36)	ELISA	Healthy volunteers
Akhlaghi [43]	2005	Kordestan	1114	37	3.32 (2.27, 4.37)	IFA	Healthy volunteers
Rafiei [20]	2007	Khuzestan	3446	475	13.78 (12.63, 14.94)	ELISA	Healthy volunteers
Baharsefat [34]	2007	Golestan	1024	46	4.49 (3.22, 5.76)	ELISA, IFA	Healthy volunteers
Mirzanejadasl [30]	2008	Ardabil	1003	111	11.07 (9.13, 13.01)	ELISA	Healthy volunteers
Hadadian [23]	2008	Kordestan	1979	22	1.11 (0.65, 1.57)	ELISA	Healthy volunteers
Moazezi [31]	2009	Kerman	451	37	8.20 (5.67, 10.74)	ELISA	Blood donors
Akhlaghi [44]	2009	Tehran	1100	18	1.64 (0.01, 0.02)	Dot-ELISA	Healthy volunteers
Esmaeili [45]	2010	Kashan	361	11	3.05 (0.01, 0.05)	ELISA, IFA	Healthy volunteers
Srakari [24]	2010	Yasuj	500	36	7.20 (0.05, 0.09)	ELISA	Patients referred to lab
Dadkhah [46]	2011	East Azarbaijan	250	8	3.20 (0.01, 0.05)	IFA	Healthy volunteers
Harandi [35]	2011	Kerman	1140	34	2.98 (0.02, 0.04)	ELISA	Healthy volunteers
Kavous [25]	2010	Jahrom	1096	69	6.30 (0.05, 0.08)	ELISA	Patients referred to lab
Garedaghi [47]	2011	East Azarbaijan	1500	11	0.73 (0, 0.01)	ELISA	Healthy volunteers
Heidari [26]	2011	Ardabil	670	12	1.79 (0.01, 0.03)	ELISA	Healthy volunteers
Rakhshanpour [36]	2012	Qom	1564	25	1.60 (0.01, 0.02)	ELISA	Healthy volunteers
Zibaei [16]	2013	Khorram abad	617	95	15.40 (0.13, 0.18)	ELISA	Patients referred to lab
Asgari [19]	2013	Arak	578	20	3.46 (0.02, 0.05)	ELISA	Healthy volunteers
Shahrokhabadi [48]	2014	Kerman	486	9	1.85 (0.01, 0.03)	ELISA	Patients referred to HC
Ilbeigi [33]	2015	Isfahan	635	7	1.10 (0, 0.02)	ELISA	Patients referred to HC

*N*: number of positive; HC: health center.

**Table 2:** Subgroup meta-analysis of cystic echinococcosis for age, sex, and lab methods.

Variable	Number	Prevalence (%)	95% CI		*I* ^2^	*Q*-test	*p*
			Lower	Upper			
Age (year)							
<30	7	2.2	1.9	3.3	93.2%	862.8	*p* < 0.001
30–40	9	2.8	2.1	4.2	94.5%		
>40	6	4.1	3.0	4.8	98.7%		

Sex							
Male	21	2.1	1.8	2.4	94.9%	398.1	*p* < 0.001
Female	10	3.6	3.2	3.9	96.2%		

Lab methods							
CIE	3	6.0	5.3	6.7	96.6%	720.9	*p* < 0.001
ELISA	15	3.2	3.0	3.4	98.6%		
IFA	9	2.2	1.8	2.5	97.4%		
ELISA & Others	4	1.8	1.5	2.1	91.8%		
